# The development and evaluation of a five-language multi-perspective standardised measure: clinical decision-making involvement and satisfaction (CDIS)

**DOI:** 10.1186/1472-6963-14-323

**Published:** 2014-07-28

**Authors:** Mike Slade, Harriet Jordan, Eleanor Clarke, Paul Williams, Helena Kaliniecka, Katrin Arnold, Andrea Fiorillo, Domenico Giacco, Mario Luciano, Anikó Égerházi, Marietta Nagy, Malene Krogsgaard Bording, Helle Østermark Sørensen, Wulf Rössler, Wolfram Kawohl, Bernd Puschner

**Affiliations:** 1 Section for Recovery (Box P029), Institute of Psychiatry, King’s College London, De Crespigny Park, London SE5 8AF, UK; 2 Department of Psychiatry and Psychotherapy II, Section Process-Outcome Research, Ulm University, Ludwig-Heilmeyer-Str. 2, Günzburg 89312, Germany; 3 Department of Psychiatry, University of Naples SUN, Largo Madonna delle Grazie, Naples 80138, Italy; 4 Medical and Health Science Center, Department of Psychiatry, University of Debrecen, Nagyerdei krt. 98, Debrecen 4012, Hungary; 5 Unit for Psychiatric Research, Aalborg Psychiatric Hospital, Aarhus University Hospital, Mølleparkvej 10, Aalborg 9000, Denmark; 6 Department of General and Social Psychiatry, University of Zurich, Militärstrasse 8, Zurich 8021, Switzerland

**Keywords:** Clinical decision-making, Involvement, Satisfaction, Mental health, Psychometric, Translation, Patient reported outcome measure, Recovery

## Abstract

**Background:**

The aim of this study was to develop and evaluate a brief quantitative five-language measure of involvement and satisfaction in clinical decision-making (CDIS) – with versions for patients (CDIS-P) and staff (CDIS-S) – for use in mental health services.

**Methods:**

An English CDIS was developed by reviewing existing measures, focus groups, semistructured interviews and piloting. Translations into Danish, German, Hungarian and Italian followed the International Society for Pharmacoeconomics and Outcomes Research (ISPOR) Task Force principles of good practice for translation and cultural adaptation. Psychometricevaluation involved testing the measure in secondary mental health services in Aalborg, Debrecen, London, Naples, Ulm and Zurich.

**Results:**

After appraising 14 measures, the Control Preference Scale and Satisfaction With Decision-making English-language scales were modified and evaluated in interviews (n = 9), focus groups (n = 22) and piloting (n = 16). Translations were validated through focus groups (n = 38) and piloting (n = 61). A total of 443 service users and 403 paired staff completed CDIS. The Satisfaction sub-scale had internal consistency of 0.89 (0.86-0.89 after item-level deletion) for staff and 0.90 (0.87-0.90) for service users, both continuous and categorical (utility) versions were associated with symptomatology and both staff-rated and service userrated therapeutic alliance (showing convergent validity), and not with social disability (showing divergent validity), and satisfaction predicted staff-rated (OR 2.43, 95%CI 1.54- 3.83 continuous, OR 5.77, 95%CI 1.90-17.53 utility) and service user-rated (OR 2.21, 95%CI 1.51-3.23 continuous, OR 3.13, 95%CI 1.10-8.94 utility) decision implementation two months later. The Involvement sub-scale had appropriate distribution and no floor or ceiling effects, was associated with stage of recovery, functioning and quality of life (staff only) (showing convergent validity), and not with symptomatology or social disability (showing divergent validity), and staff-rated passive involvement by the service user predicted implementation (OR 3.55, 95%CI 1.53-8.24). Relationships remained after adjusting for clustering by staff.

**Conclusions:**

CDIS demonstrates adequate internal consistency, no evidence of item redundancy, appropriate distribution, and face, content, convergent, divergent and predictive validity. It can be recommended for research and clinical use. CDIS-P and CDIS-S in all 3 five languages can be downloaded at http://www.cedar-net.eu/instruments.

**Trial registration:**

ISRCTN75841675.

## Background

All clinical care results from a series of decisions made by staff and service users. Decision making is a complex and dynamic social interaction [1]. The balance of involvement between staff and service user can be conceptualised as a continuum from paternalistic or passive, (decision is made by the staff, service user consents) through shared (information is shared and decision jointly made) to informed or active (staff informs, service user decides) [2].

The optimal decision-making style varies across individuals and decision types [3]. Influences might include level of preference for information, existence of available treatment options, involvement in shared decision making, and decisions which are more values-based (i.e. where clinical equipoise exists) versus those that where there is a clearly superior treatment option. Empirical evidence from physical health settings suggests that shared decision making leads to better outcomes, including help-seeking behaviour [4], increased compliance with decisions [5], reduction in errors [6], reduced stigma and increased involvement [7]. Shared decision making involves clinician and patient as active agents in the decision making process, with both bringing information and values into the discussion, evaluating the options and taking steps to build a consensus [8]. Although shared decision making is recommended in clinical guidelines [9], the research base for SDM in mental health settings is limited. A Cochrane review of shared decision making in mental health concluded that there was insufficient evidence to draw firm conclusions, and highlighted an “*urgent need for further research*” [10].

Despite this evidence base, paternalistic decision-making remains common [1]. A primary flow of information from staff to service user means that the service user’s values and treatment preferences may be given less importance [11]. This is particularly problematic in a mental health context, where a positive working relationship supports recovery [12] and where many clinical decisions relate to the broader functioning and disability issues rather than primarily to reducing pathology. Interventions are now being developed to redress this imbalance [13], but challenges remain. Perceptions about level of involvement differ, with service users identifying paternalistic and staff identifying shared approaches [14]. In common with other mental health domains such as need and therapeutic alliance [15], this indicates the importance of separately assessing staff and service user perspectives [16].

Research into satisfaction in mental health care usually looks at the overall experience, using measures of satisfaction with overall care [17,18] rather than with a specific decision. Despite the increasing availability of decision-making measures [19], there remains a need for a short standardised measure of involvement and satisfaction with a specific decision, which is suitable for use across a range of clinical settings and countries [20].

The aim of this study was to develop and evaluate a quantitative measure of involvement and satisfaction with a specific clinical decision, with staff-rated and service user-rated versions each in five languages (Danish, English, German, Hungarian and Italian). The measure was called Clinical Decision-making Involvement and Satisfaction (CDIS). Five principles were used to inform the development of CDIS:

1. In line with research into other subjective constructs [21,22], there are likely to be differing perspectives between staff and service users, so separate assessments for use by staff (CDIS-S) and service users/patients (CDIS-P) are needed.

2. Since involvement and satisfaction can vary for different decisions even within the same meeting, the rating is made in relation to a single decision

3. Parochial references to a particular professional group, or a style or setting of working, are to be avoided to minimise country-specific items which reduce cross-cultural validity.

4. The measure should be as brief and easy to use as possible, to maximise its utility for both research and routine clinical use.

5. CDIS should as far as possible be based on existing standardised measures.

## Methods

### Design

The study comprised three stages. Stage 1 (Development of source language CDIS) involved literature review of existing standardised measures, focus groups, semi-structured individual interviews and draft measure development. Stage 2 (Development of target language CDIS) was based on the International Society for Pharmacoeconomics and Outcomes Research (ISPOR) Task Force principles of good practice for the translation and cultural adaptation of patient-reported outcome measures [23]. The ISPOR Framework identifies ten sequential steps: 1 preparation; 2 forward translation; 3 reconciliation; 4 back translation; 5 back translation review; 6 harmonisation; 7 cognitive debriefing; 8 review of cognitive debriefing results and finalisation; 9 proof-reading; and 10 final report. We refer to these steps as ISPOR 1 to ISPOR 10 respectively. Finally, Stage 3 (Psychometric evaluation) investigated stability and validity across all six sites.

### Setting

Sites in six European countries participated: Ulm University, Germany (coordinating centre for the study); Institute of Psychiatry, King’s College London, England (lead for this sub-study); University of Naples SUN, Italy; Aalborg Psychiatric Hospital, Denmark; Debrecen University, Hungary; and University of Zurich, Switzerland. Fuller details on each site and the overall study in which this study was nested are published elsewhere [2]. The study protocol was approved by ethical committees in all six sites: Ulm University Ethics Commission; Joint South London and Maudsley and Institute of Psychiatry Research Ethics Committee; Ethical Committee of the Second University of Naples, Naples; National Committee on Health Research Ethics, North Denmark Region; Regional and Institutional Ethics Committee, University of Debrecen Medical and Health Science Center; Kantonale Ethikkommission Zürich. Informed consent was obtained from all service user participants.

### Sample

Service user participants for Stages 1 and 2 were convenience samples of native speaker adults aged 18–60 using local community-based non-forensic secondary mental health services. Staff participants worked in these services. For Stage 3, inclusion criteria for service user participants in the cohort study were: aged 18–60; sufficient command of the local language; having a primary research diagnosis of mental disorder other than learning disability, dementia, substance abuse or organic brain disorder established using Structured Clinical Interview for DSM-IV (SCID) [24]; cognitive ability to give informed consent and complete study measures; expected contact with services during the study period; and presence of a severe mental illness for at least two years. Severity was tested using the Threshold Assessment Grid (TAG) [25], a measure of mental health problem severity with adequate psychometrics [26] and feasibility [27], and for which a score of 5 or more (range 0 to 24) was used as an inclusion criterion as it indicates mental illness severity sufficient to warrant specialist mental health care [28]. A paired member of staff was identified by the service user.

### Measures

The topic guides for Stage 1 individual interviews in England and focus groups in Germany were developed by the local researchers, and explored the conceptual understanding of clinical decision-making. Topics covered included experience of making decisions, and level of involvement and satisfaction with the process. The topic guides for Stage 2 focus groups incorporated the conceptual questions developed during Stage 1, along with discussion of the draft CDIS in relation to comprehensibility, aspects to improve and feasibility.

The **Feasibility Questionnaire** is a 6-item respondent-rated study-specific measure assessing feasibility [29], covering length, conceptual comprehensibility, language comprehensibility, acceptability, and conceptual coverage of involvement and satisfaction. This approach has been used to investigate the feasibility of other measures [30,31]. Each item is rated from 0 (worst) to 4, and feasibility is adequate if the mean rating is more than 2 for each item.

Three staff-rated assessments were used to assess validity in Stage 3 (Psychometric evaluation). The Global Assessment of Functioning (**GAF**) is a one-item global measure of symptomatology and social functioning, with a scale ranging from 1 (worst) to 99 (best) [32]. The Health of the Nation Outcome Scales (**HoNOS**) is a 12-item assessment of social disability, with a summary score ranging from 0 (worst) to 48 (best) [33]. The Helping Alliance Scale – Staff (**HAS-S**) is a five-item measure of therapeutic alliance, with a summary score ranging from 0 (worst) to 10 (best) [34]. Specific HAS-S items used in this study were item 4 (“Do you feel you are actively involved in the treatment of the service user?”) and item 5 (“Do you feel you can help and effectively treat the service user?”).

Four service user-rated assessments were used to assess validity in Stage 3 (Psychometric evaluation). The Outcome Questionnaire-45 (**OQ-45**) is a 45-item measure of symptomatology, with a total score (TOT) ranging from 0 (best) to 180 (worst) [35]. The HAS – Patient (**HAS-P**) is a six-item measure of therapeutic alliance, with a summary score ranging from 0 (worst) to 4 (best) [34]. Specific HAS-P items used in this study were item 4 (“Is your staff member committed to and actively involved in your treatment?”) and item 6 (“How do you feel immediately after a session with your staff member?”). The Manchester Short Assessment (**MANSA**) is a 12-item measure of quality of life, with a summary score ranging from 1 (worst) to 7 [36]. The 30-item version of the Stages of Recovery Inventory (**STORI**) allocates participants to one of five stages of recovery. Because the original psychometric study [37] and two replication studies [38,39] all identified a 3-cluster solution better fitted the data, the summary score was allocation to one of three stages: Moratorium, Awakening/Preparation, Rebuilding/Growth.

### Procedures

#### Stage 1 (Development of source language CDIS)

Three sources of data were used to develop the draft CDIS in English from February 2009 to May 2009. First, a non-systematic scoping review [40] was undertaken to identify existing standardised self-rated English-language measures of involvement and satisfaction in clinical decision-making from both service-user and staff perspectives. The Web of Knowledge database was searched using the terms: measures, decision making, satisfaction, mental illness, shared decision making, mental health care, and decision making involvement. Key psychometric properties were rated independently by two raters using categories shown in Table 1, with disagreements resolved through team discussion. Permission was sought from the authors of measures which were to be modified for use in CDIS. Consideration was given to inviting the instrument developer to be involved in the translation process, but no measure was identified which overlapped sufficiently with the intended focus and use of CDIS, so no instrument developer was involved beyond giving permission.

**Table 1:** Summary of psychometric properties of measures (n = 8)

**Measure**	**Content validity**	**Construct validity**	**Floor/Ceiling effect**	**Internal consistency**	**Reliability**	**Brevity**	**Simplicity**	**Relevance**
**Measures of involvement only**								
Patients Preference for Control [41]	Unknown	Unknown	Unknown	Unknown	Unknown	Adequate	Adequate	Adequate
Control Preferences Scale [42]	Unknown	Adequate	Unknown	Unknown	Adequate	Adequate	Adequate	Adequate
Facilitation of Patient Involvement [43]	Unknown	Adequate	Unknown	Adequate	Adequate	Adequate	Adequate	Doubtful
Desire to Participate in Medical Decision Making Scale [44]	Adequate	Doubtful	Unknown	Adequate	Adequate	Adequate	Adequate	Doubtful
Decision Self-Efficacy Scale [45]	Unknown	Adequate	Unknown	Adequate	Adequate	Adequate	Adequate	Poor
**Measures of satisfaction only**								
Satisfaction with Decision Scale [46]	Unknown	Adequate	Unknown	Adequate	Adequate	Adequate	Adequate	Adequate
**Measures of involvement and satisfaction**								
Decisional Conflict Scale [47]	Unknown	Adequate	Unknown	Adequate	Adequate	Adequate	Adequate	Poor
Autonomy Preference Index [48]	Adequate	Adequate	Poor	Adequate	Adequate	Poor	Doubtful	Doubtful

Second, individual interviews about the concept of clinical decision-making were undertaken in England with a convenience sample of staff and service users. The topic guide asked about types of decision, level of involvement and satisfaction experienced, and approaches to decision-making. Service user participants were paid £5 for their involvement.

Third, focus groups about the concept of clinical decision making were undertaken in Germany with a convenience sample of service users [49]. Data were collected in Germany to provide a comparison with the data from England, so as to identify culturally-specific aspects which were less applicable for use in the measure. Participants were paid €10 for their involvement.

On the basis of these three sources of data, a draft English CDIS was developed in English (the ‘source’ language) with two versions: service user-rated CDIS-P and staff-rated CDIS-S. This was then evaluated in England with a further focus groups with service users and staff (topic guide: decision-making, comments on draft CDIS), modified and then piloted with both staff and service users (completing CDIS and Feasibility Questionnaire). The draft CDIS was modified to produce the final English CDIS.

#### Stage 2 (Development of target language CDIS)

##### ISPOR Stages 1 to 3: forward translation and reconciliation

All ten stages of the ISPOR principles were used. Preparation (ISPOR stage 1) was undertaken at a study meeting involving researchers from all six study sites held in Ulm in May 2009. A forward translation (ISPOR stage 2) of CDIS into the four ‘target’ languages (Danish, German, Hungarian and Italian) was made by bilingual translators in each country who were native speakers in the target language. Consideration was given to producing multiple forward translations to minimise the impact of an individual’s writing style on the translation, but this proved unnecessary as the translation task was relatively straightforward and the ISPOR guidelines indicated low agreement on how multiple forward translations are reconciled into one final version. In order to maximise the conceptual equivalence of the draft CDIS, a staff focus group and a service user focus group were held in all six countries. Reconciliation (ISPOR stage 3) comprised careful review of the forward translation and the results from the focus groups by each site to ensure conceptual equivalence with the aims of the measure discussed at the Ulm meeting.

### ISPOR stages 4 to 5: back translation

A back translation (ISPOR stage 4) of each translated version was then made by a different bilingual translator into English, without knowledge of the original English version. Back-translation is a quality control step to demonstrate that the target language version does not have a different content or conceptual basis which would compromise psychometric properties and reduce data quality. As the constructs being assessed were subjective, a focus on conceptual rather than literal translation was used.

The back translation review (ISPOR stage 5) was undertaken by researchers at the English site. Reconciliation to ensure the conceptual equivalence of the translation involved identification of discrepancies between the original English language version and the back translation, and refinement of the target language versions. The aim was to minimise mistranslation or omission.

### ISPOR stages 6 to 10: harmonisation and review

Following harmonisation (ISPOR stage 6) of all target language translations on the basis of back translations, the source and all four target language versions of CDIS were piloted using cognitive debriefing (ISPOR stage 7) in each country. The aim was to assess the level of comprehensibility and cognitive equivalence of the translations, and to highlight items that may be inappropriate at a conceptual level. The ISPOR guidance indicates that testing should involve five to eight respondents who are native speakers of the target language and represent the target population in clinical and sociodemographic characteristics. Therefore piloting was undertaken with community-based non-forensic secondary adult mental service users and associated staff in each country (including England). Participants were paid £10 or the local equivalent for their involvement in some sites.

Finally, a review of the cognitive debriefing and finalisation of all new translations (ISPOR stage 8) was completed at a study meeting involving researchers from all six study sites, held in Zurich in September 2009. Following careful proof-reading (ISPOR stage 9) by all sites, this produced agreement on the final CDIS with staff and service user versions in five languages. This paper comprises the final report (ISPOR stage 10) of the process, along with the final report to be submitted to the study funders when the study has concluded.

#### Stage 3 (Psychometric evaluation)

Psychometric properties were investigated using data collected in a six-country cohort study. A cohort of service users with TAG score of 5 or more (indicating more severe mental illness) was identified and recruited between November 2009 and November 2010 in each site. Service users identified a member of staff whom they saw regularly, and then identified a specific decision made at their last meeting (generally within the last two weeks). A decision was defined as a topic which was (a) discussed, with the result that (b) either changes were made or there was agreement that no changes should be made. The service user then completed CDIS-P in relation to that decision, HAS-P in relation to the nominated staff member, OQ-45, MANSA and STORI. Their nominated staff member was informed of the decision and asked to complete CDIS-S, HAS-S, HoNOS and GAF. Research diagnosis was established by the researchers using SCID from clinical notes. Service users were paid £20 (or local equivalent) and staff were paid £10 for their involvement (which included completion of other measures not reported here) in some sites. Two months later, service users and staff were asked whether they had implemented the decision (Yes, Partly or No). Service users were paid £5 for their involvement in some sites.

Data from all sites were electronically collated into a central database, with data cleaning led by the co-ordinating centre. Cleaning involved data validation and data verification. Data validation involved (i) checking the case-level data were internally consistent, and (ii) identifying outlier ratings, asking the originating site to manually check each identified outlier rating against paper and local electronic databases, and correcting the central database where necessary. Data verification involved identifying remaining outliers and deciding whether to include them in the analysis on the basis of plausibility, *i.e.* whether they were reasonable ratings and whether they correlated with other contemporaneous ratings for the same participant.

### Analysis

All focus groups and interviews were recorded and transcribed into the local language. For the focus groups, consideration was given to translating transcripts into English and then back-translating to validate the transcript prior to analysis of the aggregated English transcripts. This approach was not used because the qualitative aspect of the study was not focussed on developing an overall conceptual understanding of clinical decision-making across all sites. Rather, the aim of all focus groups was more local - either to provide data relating specifically to the local language version of CDIS or to provide a thematic overview of the conceptual meaning of clinical decision-making in each site, so as to ensure broad conceptual equivalence. Therefore thematic analysis of both interviews and focus groups was undertaken locally, without translation into English. This involved the development by two independent analysts of an initial coding framework capturing the overarching and related sub-themes within the local language transcript, which was then synthesised through discussion with modification or addition of codes until theoretical saturation was obtained. An English version of the coding framework was generated by local translation of the coding framework into English, which was then reviewed by a native English speaker and modified by the local site if necessary.

The investigation of internal consistency used Cronbach’s alpha, with a score between 0.70 and 0.95 indicating good internal consistency [50]. A higher alpha was acceptable because for shorter scales (such as CDIS) all items may be clinically informative rather than indicating item redundancy. Item-level deletion was use to investigate whether removal of any item would markedly improve internal consistency. Floor and ceiling effects are particularly important with CDIS since the aim is to compare between groups, and such effects may make it impossible to determine the central tendency and hence detect difference. Therefore the distribution across the range of scores was investigated, with normal distribution indicating minimal floor or ceiling effects. Construct validity was investigated in two ways. First, convergent validity was investigated by testing relationships assumed to co-vary. CDIS Involvement was expected to relate specifically to stage of recovery (STORI), and also to functioning (GAF) and subjective well-being (MANSA). CDIS Satisfaction was expected to relate to the relationship (HAS-S and HAS-P) and symptom distress (OQ-45 symptom distress sub-scale). Second, divergent validity was investigated by testing relationships assumed not to correlate: CDIS and symptoms (OQ-45 symptom distress sub-scale) and social disability (HoNOS). The ordinal STORI analysis involved cross-tabulation with STORI category, ordinal logistic regression to estimate the probability of participants being in a less active CDIS category with lowest recovery stage (Moratorium) as reference category, and Wald test to test null hypothesis of no difference in odds ratio of being in a less active CDIS category. Other variables were continuous, so bivariate relationships were assessed using Spearman's Rank correlation. Following these analyses, adjustment was made for staff rating more than one service user. For CDIS Involvement and the categorical CDIS Satisfaction (utility), univariable ordinal logistic regression models were used including a random effect to adjust for clustering by staff, with results reported as odds ratios showing the odds of being in a higher CDIS category. For the continuous CDIS Satisfaction, univariable linear regression models including a random effect to adjust for clustering by staff and with resampling using bootstrapping (5000 repetitions) was used. Predictive validity was analysed by comparing satisfaction and involvement with ratings by the same rater (staff/service user) of implementation of the decision (Yes vs. Partly vs. No) made 2 months later. Satisfaction was expected to predict implementation, whereas involvement was not (since no *a priori* stance was taken in this study about the relative merits of different involvement experiences). Ordinal regression models were estimated with a random effect to adjust for clustering by staff. For categorical predictors (Involvement and Satisfaction (Utility)), odds ratios show the estimated odds of being in a higher implementation category for this category as compared to the reference category (Active involvement and Low satisfaction respectively). For continuous predictors (Satisfaction), odds ratios show the estimated odds of being in a higher implementation category for every one unit increase in predictor. All quantitative analyses were undertaken using SPSS 19.0 and Stata 11.2.

## Results

### Stage 1 (Development of source language CDIS)

The literature review identified 218 papers. Titles and abstracts were reviewed, identifying 14 measures. The relevant articles and measures were obtained and reviewed. Six measures were excluded as they assessed satisfaction with more general aspects of care [51-55] or were not self-rated [56]. The psychometric properties for the remaining eight measures are shown in Table 1.

Two measures provided the strongest evidence of psychometric properties. The Control Preference Scale (**CPS**) is a single-item patient-rated measure of preferred style of involvement [42]. The scale comprises Active (“I prefer to make the final selection about which treatment I will receive”, “I prefer to make the final selection of my treatment after seriously considering my doctor’s opinion”), to Collaborative (“I prefer that my doctor and I share responsibility for deciding which treatment is best for me”) and Passive (“I prefer that my doctor make the final decision about which treatment will be used, but seriously consider my opinion”, “I prefer to leave all decisions about my treatment to my doctor”). It was initially developed for use in cancer patients, but has been adapted and used with mental health populations [57,58]. The Satisfaction With Decision-making (**SWD**) scale is a 6-item patient-rated measure of satisfaction [46]. The items cover adequacy of supplied information, was it the best decision, consistency with personal values, expectation of full implementation, whether it was my decision, and overall satisfaction. The five-point scale ranges from Strongly Disagree to Strongly Agree. SWD was originally developed in the context of postmenopausal hormone-replacement therapy decisions [46], and has been validated for use with people with depression [59].

Interviews about clinical decision-making were held with four service users (age 33–46, 3 female, all psychosis diagnosis) and five staff (nurse, clinical psychologist, psychiatrist, occupational therapist, educator). A range of types of decision were identified: most commonly medication and psychological treatments, but also for example pre-conception counselling, diet, housing, benefits, structuring time, involvement of a relative, and employment. Influences on satisfaction identified by service users were level of choice, preferences being respected, setting the agenda for the conversation, saying what I want to say, and the relationship with staff. For staff, influences on their satisfaction were the relationship, being empowering, role conflict (therapeutic benefit versus risk management), giving information, level of collaboration, and supporting the service user to decide. Both service users and staff highlighted the ethical and power balances involved in decision-making conversations, with one stating “*it’s more about a learning process, not all-or-none*”. There was consensus that the best outcome occurs when service user makes a decision with which both agree. These interviews informed the measure by: (i) identifying the need for the wording to be generic, rather than assuming that the decision is about treatment; (ii) identifying the influences on involvement and satisfaction differ, pointing to the need for separate staff and service user versions; and (iii) identifying that comparable versions to allow direct comparison were preferable to incompatible staff and service user versions.

Two focus groups were conducted in Germany with service users only (n = 3 and n = 5). The emergent coding framework (not reported in full) identified themes of the nature of the illness (burden, course), relationships (how staff perceive the service user, staff response to non-cooperation, how mis-communication is handled), service user characteristics (communication difficulties, how illness is understood), the nature of the decision (type, who is involved, whether implemented and why) and decision-making processes (information supplied, involvement) [49]. These findings were consistent with the London interviews.

Overall the qualitative data indicated conceptual equivalence could be achieved by modifying CPS (to measure involvement in a specific decision rather than general preference) and SWD (to modify administration instructions). A draft English CDIS was developed using these measures, with modifications in items to produce a staff-rated version. Other modifications were formatting and instructions for raters. The draft English CDIS was then evaluated in two ways. A service user focus group (n = 7, 3 female) identified that the six-item satisfaction scale looked “all the same”, commented on wording and how to identify who made the decision, and preferred the 1-item Involvement Sub-scale as clearer. The staff focus group (n = 7, 5 female, nurse/occupational therapist/social worker/support worker) contrasted team and individual staff views, wanted to record dissent when the service user makes a non-consensus decision, challenged the assumption that there is one ‘best’ decision, identified that the optimal amount of involvement in decision-making differs, and noted the absence of carer involvement. As a result, the draft English CDIS wording was modified (“I” became “We” in CDIS-S), administration instructions were made more accessible and modified to suggest the first administration is done with service user (to ensure comprehension), the Involvement sub-scale was finalised as categorical (to indicate that different points may be desirable in different situations), and a comments box was added to the staff version. Piloting of CDIS with service users (n = 9) and staff (n = 7) evaluated feasibility, finding adequate results with mean ratings on the Feasibility Questionnaire ranging for service users from 2.89 to 3.22, and for staff from 2.75 to 3.25.

The final version of the Clinical Decision-making Involvement and Satisfaction (CDIS) scale is shown in Table 2.

**Table 2:** Contents of final CDIS

**Wording in CDIS-S**	**Wording in CDIS-P**	
**Involvement sub-scale** (one item)		*Score*
The service user made the final decision	I made the final decision.	Active
The service user made the final decision after seriously considering my opinion	I made the final decision after seriously considering my clinicians opinion	Active
The service user and I shared responsibility for making the best decision for them	My clinician and I shared responsibility for making the best decision for me	Shared
I made the final decision, after seriously considered the service user’s opinion	My clinician made the final decision, but seriously considered my opinion	Passive
I made the final decision	My clinician made the final decision	Passive
**Satisfaction sub-scale** (six items)		*Rating scale*
1. I had adequate information from the service user about the issues important to them	1. I am satisfied that I am adequately informed about the issues important to the decision	5-point Likert scale from 1 (Strongly disagree) to 5 (Strongly agree)
2. The decision we made was the best decision possible in my view	2. The decision we made was the best decision possible in my view	
3. I am satisfied that the decision was consistent with my personal and professional values	3. I am satisfied that the decision was consistent with my personal values	
4. I expect the decision we made to be successfully acted on/continued to be acted on	4. I expect the decision we made to be successfully acted on/continued to be acted on	
5. I am satisfied that this was the decision to make	5. I am satisfied that this was the decision to make	
6. I am satisfied with the decision	6. I am satisfied with the decision	

CDIS is rated in relation to a specific identified decision. The Involvement sub-scale comprises one item about level of involvement experienced, which uses five categories. Categories 1 and 2 are collapsed (as their distinction may reflect social desirability bias rather than different experiences) to be scored as Active involvement, category 3 is Shared involvement, and categories 4 and 5 are collapsed to Passive involvement. Note therefore that staff-rated Passive involvement indicates passive involvement by the service user, i.e. active staff involvement. The Satisfaction sub-scale is valid if all six items are rated, and is scored as the mean of all items, ranging from 1 (low satisfaction) to 5.

### Stage 2 (Development of target language CDIS)

The draft CDIS was translated into each target language (Danish, German, Hungarian, Italian). Focus groups were then held in Naples (n = 4 service users, n = 5 staff), Aalborg (n = 3 service users, n = 4 staff), Debrecen (n = 4 service users, n = 5 staff) and Zurich (n = 6 service users, n = 7 staff). The relevant target language CDIS was modified in the light of the focus group, to maximise conceptual equivalence without compromising psychometrics. For example, the Danish translation of item 3 deleted “I am satisfied that” to increase comprehensibility of the item in Danish. A back translation into English was made, and reviewed in the London site, with a focus on conceptual equivalence and modifications to the target language CDIS made as indicated. The CDIS was then completed by a sample of service users (n = 30) and staff (n = 31) across all languages. For both groups, ratings for all Satisfaction items spanned at least four of the five possible ratings, and ratings for the Involvement item spanned at least four of the five categories, giving preliminary evidence of useability and no indication of floor or ceiling effects. Cognitive debriefing following administration identified no further modifications for any target language. The **final CDIS-S and CDIS-P** for each country were finalised in a study meeting. The development process involving the key stake-holders of staff and service users ensured adequate face validity and content validity.

### Stage 3 (Psychometric evaluation)

A total of 443 service users provided CDIS data. Service user characteristics and outcome assessments are shown in Table 3.

**Table 3:** Sociodemographic and clinical characteristics of service users (n = 443)

	**Ulm**	**London**	**Naples**	**Aalborg**	**Debrecen**	**Zurich**	**Total**
***n***	**91**	**43**	**86**	**70**	**95**	**58**	**443**
Male (%)	29 (32)	32 (74.4)	46 (53.5)	31 (44.3)	49 (51.6)	18 (31)	205 (53.7)
Age in years (s.d.)	45.6 (9.1)	45.2 (10.6)	43 (10.5)	39.8 (10.7)	45.2 (10)	40.4 (10.6)	42.5 (10.4)
Years in education (s.d.)	10.6 (1.7)	11.2 (1.9)	11.2 (1.9)	9.7 (1.1)	11.3 (1.3)	9.7 (1.4)	10.5 (1.9)
Marital status (%)							
*Unmarried*	32 (35)	37 (86)	39 (45)	41 (59)	43 (45)	28 (48)	220 (50)
*Married/co-habiting*	29 (32)	2 (4.2)	32 (37)	12 (17)	32 (34)	15 (26)	122 (28)
*Separated/divorced/widowed*	30 (33)	4 (9.4)	15 (17)	16 (23)	20 (21)	15 (26)	100 (23)
Living situation (%)							
*Alone*	50 (55)	13 (30.2)	13 (15)	40 (57)	21 (22)	36 (63)	173 (39)
*With spouse/partner*	31 (34)	2 (4.7)	35 (40)	18 (26)	34 (36)	16 (28)	136 (31)
*With others*	10 (11)	28 (65.1)	37 (43)	12 (17)	40 (42)	5 (9)	132 (30)
Employment (%)							
*Paid/student*	24 (26)	4 (9.3)	27 (31)	4 (6)	18 (19)	21 (36)	100 (23)
*Sheltered employment*	5 (6)	0	1 (1)	1 (1)	3 (3)	1 (2)	11 (2)
*None*	60 (67)	37 (86)	56 (65)	65 (92)	73 (76)	36 (63)	327 (74)
Main income (%)							
*Salary*	20 (22)	0	27 (31)	3 (4)	18 (19)	13 (23)	81 (19)
*Benefits*	20 (22)	34 (79.1)	27 (31)	20 (29)	7 (7)	3 (5)	103 (24)
*Pension*	40 (44)	0	2 (2)	45 (64)	67 (71)	35 (63)	189 (44)
*Family support*	11 (12)	2 (4.7)	38 (44)	0	1 (1)	5 (9)	57 (13)
Years since first contact with mental health services (s.d.)	14.1 (9.1)	13.1 (9.1)	12.7 (10.04)	12 (8)	13.6 (7.5)	8.7 (8.3)	12.5 (8.9)
DSM-IVR Research diagnosis							
*Schizophrenia and other psychotic disorders*	32 (35)	25 (58.2)	23 (27)	45 (64)	54 (56)	15 (26)	194 (44)
*Mood disorders*	41 (45)	5 (11.6)	33 (38)	17 (24)	20 (21)	27 (47)	143 (32)
*Other*	18 (20)	7 (16.3)	30 (35)	8 (11)	21(22)	16 (28)	98 (22)
Mental health in-patient admissions in previous year (%)	44 (48)	11 (25.6)	0	15 (21)	23 (24)	25 (43)	118 (27)
**Service user-rated outcomes**							
STORI (%)							
*Moratorium*	16 (18)	7 (16)	20 (23)	13 (19)	16 (17)	13 (22)	85 (19)
*Awakening/Preparation*	16 (18)	13 (30)	35 (21)	7 (10)	11 (12)	15 (26)	97 (22)
*Rebuilding/Growth*	58 (64)	23 (54)	31 (36)	50 (71)	68 (72)	30 (52)	260 (59)
MANSA (mean, s.d.)	4.4 (1.0)	4.2 (0.8)	3.43 (0.8)	4.6 (1)	4.5 (0.8)	3.6 (1.2)	4.1 (1.0)
OQ-45 (mean, s.d.)	77.4 (28.2)	66.1 (2.6)	77.2 (22.7)	69.7 (24.7)	61.4 (23)	81.2 (26.7)	72.2 (25.9)
HAS-P (mean, s.d.)	7.1 (1.3)	6.8 (2.4)	7.3 (1)	7.2 (1.3)	7.7 (0.7)	8.1 (1.3)	7.2 (1.3)
**Staff-rated outcomes**							
TAG (mean, s.d.)	8.2 (2.3)	8.9 (2.2)	7.6 (2.4)	7.4 (2.2)	6.3 (1.6)	6.7 (1.8)	7.4 (2.2)
HAS-S (mean, s.d.)	7.6 (1.0)	8.1 (1.0)	8.1 (1.7)	8.1 (1.2)	7.9 (1.1)	8.2 (0.8)	8.0 (102)

152 staff provided complete CDIS data for 405 (91%) of these 443 service users (pooled sample 45% male, mean age 44.0 years, 54% psychiatrist/36% care co-ordinator/7% psychologist/3% social worker, mean 15.3 years work experience).

### Stability

The distribution of CDIS scores across sites is shown in Table 4.

**Table 4:** CDIS Service user (n = 443) and staff (n = 405) ratings by site

	**Ulm**	**London**	**Naples**	**Aalborg**	**Debrecen**	**Zurich**	**Total**
**CDIS SERVICE USER *n***	***91***	***43***	***86***	***70***	***95***	***58***	***443***
**Satisfaction** mean (s.d.)	4.11 (0.70)	4.21 (0.92)	4.25 (0.71)	4.45 (0.53)	4.45 (0.56)	3.75 (0.58)	4.23 (0.69)
**Involvement** n (%)							
Passive involvement	28 (31)	8 (19)	25 (29)	16 (23)	27 (28)	14 (24)	118 (26.6)
Shared involvement	36 (40)	13 (30)	50 (58)	33 (47)	57 (60)	30 (52)	219 (49.4)
Active involvement	27 (30)	22 (51)	11 (13)	21 (30)	11 (12)	14 (24)	106 (23.9)
**CDIS STAFF *n***	*80*	*23*	*86*	*67*	*95*	*54*	*405*
**Satisfaction** mean (s.d.)	3.94 (0.53)	4.24 (0.54)	4.32 (0.58)	4.09 (0.65)	4.22 (0.61)	4.02 (0.42)	4.14 (0.58)
**Involvement** n (%)							
Passive involvement	13 (16)	1 (4)	55 (64)	8 (12)	9 (9)	8 (15)	94 (23.3)
Shared involvement	32 (40)	9 (39)	27 (31)	23 (34)	64 (67)	32 (59)	187 (46.3)
Active involvement	34 (43)	13 (57)	4 (5)	36 (54)	22 (23)	14 (26)	123 (30.4)

For staff, CDIS Satisfaction sub-scale (rated for 403 service users) internal consistency was 0.89, internal consistency after item-level deletion ranged from 0.86 to 0.89, and distribution of scores across the range was 1% 1.0-2.0, 3% 2.1-3.0, 48% 3.1-4.0 and 43% 4.1-5.0. CDIS Involvement sub-scale (rated for 404 service users) distribution comprised Passive involvement n = 94 (23.3%), Shared involvement n = 187 (46.3%) and Active involvement n = 123 (30.4%).

For service users, CDIS Satisfaction sub-scale internal consistency was 0.90, internal consistency after item-level deletion ranged from 0.87 to 0.90, and distribution of scores across the range was 1% 1.0-2.0, 5% 2.1-3.0, 42% 3.1-4.0 and 52% 4.1-5.0. CDIS Involvement (rated by 443 service users) comprised Passive involvement n = 118 (26.6%), Shared involvement n = 219 (49.4%) and Active involvement n = 106 (23.9%).

In summary, for the Involvement sub-scale, there was appropriate distribution variation across sites as would be anticipated from cultural differences, with no indication of floor or ceiling effects.

For the Satisfaction sub-scale there was good evidence for internal consistency, with no indication of item redundancy. Distribution was right-skewed as is typical with satisfaction data. The validity of analysing CDIS Satisfaction as a collapsed ordinal scale was therefore investigated. Categories were formulated on the basis of utility where an emphasis was placed on separating categories according to clinical meaningfulness. Participants with extremely low satisfaction (rating satisfaction items as ‘Strongly disagree’) transitioning to low satisfaction (mostly rating items as ‘Disagree’) or towards moderate (mostly rating ‘Neither disagree nor agree’) would indicate a marginal improvement but remain an unsatisfactory endpoint. The ‘moderate satisfaction’ category comprised participants rating the majority of satisfaction items as ‘Agree’ with some items neutral, and high satisfaction captured participants recording almost or every satisfaction item as ‘Strongly agree’. These categories of low (1.0-3.0), moderate (3.01-4.0) and high (4.01-5.0) satisfaction were assigned uniformly distanced (ordinal) utilities of 0, 1 and 2 respectively. The categories therefore distinguish groups by their ordinal nature but not by a specific value assigned to each category. Transitions from low to moderate satisfaction and from low to high satisfaction are of primary clinical interest. The transition from moderate to high satisfaction is useful as a clinical target which makes use of the positive skew that is characteristic of satisfaction data. Distribution was more balanced than for the continuous rating: staff 17 (4.2%) low, 204 (50.5%) moderate, 183 (45.3%) high, and service users 27 (6.1%) low, 184 (41.5%) moderate, 232 (52.4%) high. CDIS Satisfaction was subsequently analysed both as continuous CDIS Satisfaction and categorical CDIS Satisfaction (Utility).

### Construct validity

CDIS Involvement (n = 403) was investigated using STORI stage. Cross-tabulation indicated staff rated more active involvement for Rebuilding and Growth (highest stage) service users (82 (34%) Active, 119 (50%) Shared, 39 (16%) Passive) than Awareness and Preparation (middle stage) (22 (26%) vs. 33 (38%) vs. 31 (36%)) and Moratorium (lowest stage) (18 (23%) vs. 35 (45%) vs. 24 (31%)). Ordinal logistic regression indicated no difference in involvement category between middle and lowest stage of recovery (OR 1.1, 95%CI 0.60 to 1.96, p = 0.78) and a significant difference between highest and lowest stage of recovery (OR 0.52, 95%CI 0.32 to 0.84, p < 0.05). The odds of being in a higher involvement category was found to be significantly different between the middle and highest stages of recovery as tested by a Wald test on the two parameters from the model (Chi^2^ = 9.39, p = 0.002). Staff rate higher CDIS Involvement for more recovered service users.

For service users, cross-tabulation indicated more active involvement for highest stage service users (69 (27%) vs. 129 (50%) vs. 62 (24%)) than middle stage (22 (23%) vs. 43 (44%) vs. 32 (33%)) and lowest stage (15 (18%) vs. 46 (54%) vs. 24 (28%)). Despite this trend towards a larger proportion of more recovered service users being actively involved (27% vs. 18%), this difference was not significant between middle and lowest stage (OR = 1.0, 95%CI 0.58 to 1.74, p = 0.97) or between highest and lowest stage (OR = 0.71, 95%CI 0.45 to 1.12, p = 0.14). The odds of being in a higher involvement category was not found to differ significantly between the middle and highest stages of recovery as tested by a Wald test on the two parameters from the model (Chi^2^ = 2.43, p = 0.12). Service user rating of involvement was not significantly higher for more recovered service users.

Convergent and divergent validity were investigated. Unadjusted correlations are shown in Table 5. (Lower Involvement score means more active involvement of the service user).

**Table 5:** Convergent and divergent validity of CDIS sub-scales (unadjusted non-parametric correlations)

	**Convergent validity**					**Divergent validity**	
	**GAF**	**MANSA**	**HAS-S**	**HAS-P**	**OQ-45 symptom distress**	**OQ-45 symptom distress**	**HoNOS**
**Staff**							
CDIS Involvement	**-0.20**	**-0.19**				0.08	0.001
	*p < 0.001*	*p < 0.001*				*p = 0.10*	*p = 0.98*
CDIS Satisfaction			**0.46**	**0.33**	**-0.15**		-0.08
			*p < 0.001*	*p < 0.001*	*p = 0.002*		*p = 0.13*
CDIS Satisfaction (Utility)			**0.38**	**0.30**	**-0.13**		**-0.11**
			*p < 0.001*	p < 0.001	*p = 0.009*		*p = 0.03*
**Service user**							
CDIS Involvement	-0.05	0.02				0.05	-0.008
	*p = 0.32*	*p = 0.70*				*p = 0.32*	*p = 0.87*
CDIS Satisfaction			**0.22**	**0.42**	**-0.24**		-0.02
			*p < 0.001*	*p < 0.001*	*p < 0.001*		*p = 0.87*
CDIS Satisfaction (Utility)			**0.19**	**0.36**	**-0.19**		-0.02
			*p < 0.001*	*p < 0.001*	*p < 0.001*		*p = 0.72*

Bold p < 0.05.

For staff ratings, convergent and divergent validity were demonstrated: staff identified more involvement from service users in later stages of recovery and with higher functioning and better quality of life, no association between involvement and either symptomatology or social disability, and more satisfaction when staff-rated and service user-rated therapeutic alliance were better and symptom distress was low. For service users, the picture was more mixed. There was no association between involvement and any other variable, and satisfaction was associated with both perspectives on therapeutic alliance. Overall, convergent validity was demonstrated for both versions of the Satisfaction sub-scale and the staff-rated Involvement sub-scale, and divergent validity was demonstrated for both sub-scales and both perspectives.

Some staff rated the same service user. The results of investigating convergent and divergent validity with adjustment for staff clustering is shown in Table 6.

**Table 6:** Convergent and divergent validity of adjusted CDIS sub-scales (adjusted regression models)

	**Convergent validity**					**Divergent validity**	
	**GAF**	**MANSA**	**HAS-S**	**HAS-P**	**OQ-45 symptom distress**	**OQ-45 symptom distress**	**HoNOS**
**Staff**							
CDIS Involvement	**0.98**	0.78				1.01	1.01
	*p = 0.048*	*p = 0.085*				*p = 0.238*	*p = 0.817*
CDIS Satisfaction			**0.18**	**0.08**	**-0.006**		-0.003
			*p < 0.001*	*p < 0.001*	*p < 0.001*		*p = 0.450*
CDIS Satisfaction (Utility)			**1.95**	**1.43**	**0.98**		0.97
			*p < 0.001*	*p < 0.001*	*p = 0.006*		p = 0.115
**Service user**							
CDIS Involvement	0.99	0.98				1.01	-0.008
	*p = 0.34*	*p = 0.84*				*p = 0.137*	*p = 0.87*
CDIS Satisfaction			**0.11**	**0.15**	**-0.008**		-0.008
			*p = 0.001*	*p < 0.001*	*p < 0.001*		*p = 0.276*
CDIS Satisfaction (Utility)			**1.35**	**1.59**	**0.98**		0.98
			*p = 0.004*	*p < 0.001*	*p < 0.001*		*p = 0.248*

Bold p < 0.05.

To aid interpretation of Table 6, staff ratings mean that for every one unit increase in GAF, the odds of being in a higher CDIS Involvement category decreases by 2%. No evidence was found of association between CDIS Involvement and MANSA, OQ-45 Symptom distress or HoNOS. For every one unit increase in HAS-S, HAS-P and OQ-45 symptom distress, CDIS Satisfaction increases by 0.18, 0.08 and -0.006 units respectively. No evidence was found for an association between CDIS Satisfaction and HoNOS. For CDIS Satisfaction (Utility), for every one unit increase in HAS-S, it is almost twice as likely to be in a higher CDIS Satisfaction utility group. For every one unit increase in HAS-P and OQ-45 Symptom distress, the odds of being in a higher CDIS Satisfaction utility group increase by 43% and decrease by 2% respectively. No evidence was found for an association between CDIS Satisfaction (Utility) and HoNOS.

Overall, these findings reflect those in the unadjusted analysis shown in Table 5, and indicate construct validity.

### Predictive validity

Predictive validity was investigated in order to show criterion-related validity. Table 7 models the relationship between involvement and satisfaction with the rating of implementation by the same rater made two months later.

**Table 7:** Predictive validity of CDIS scales for service users (n = 440) and staff (n = 402)

	**OR (95%CI)**	**SE**	**P value**
**SERVICE USERS**			
**Involvement**			
Shared vs. Active	1.54 (0.82-2,89)	0.50	0.181
Passive vs. Active	1.92 (0.94-3.94)	0.70	0.075
**Satisfaction**	**2.21 (1.51-3.23)**	0.43	<0.001
**Satisfaction (Utility)**			
Moderate vs. Low	1.40 (0.50-3.91)	0.73	0.518
High vs. Low	**3.13 (1.10-8.94)**	1.68	0.033
**STAFF**			
**Involvement**			
Shared vs. Active	**2.43 (1.31-4.50)**	0.76	0.005
Passive vs. Active	**3.55 (1.53-8.24)**	1.52	0.003
**Satisfaction**	**2.43 (1.54-3.83)**	0.57	<0.001
**Satisfaction (Utility)**			
Moderate vs. Low	**3.33 (1.11-9.97)**	1.86	0.031
High vs. Low	**5.77 (1.90-17.53)**	3.27	0.002

Bold p < 0.05.

High satisfaction predicts implementation, for both continuous and utility versions of the scale, and for both staff and service users. Active involvement is associated with lower implementation from the staff but not the service user perspective.

## Discussion and conclusions

The psychometric properties of the CDIS Scale were in general adequate. The Involvement sub-scale showed appropriate variation in distribution across sites, no floor or ceiling effects. For staff, convergent validity was shown in relation to stage of recovery, functioning and quality of life, and divergent validity in relation to symptomatology and social disability. For service users, convergent validity was not shown in relation to any considered co-variate, and divergent validity was shown in relation to symptomatology and social disability. The Satisfaction sub-scale showed internal consistency, no item redundancy, with an anticipated distribution skew towards the positive end of the scale. Convergent validity was shown in relation to staff-rated and service user-rated therapeutic alliance, and divergent validity was shown in relation to social disability. Satisfaction predicted decision implementation two months later, as did staff-rated passive involvement of the service user.

Our review identified five existing measures of involvement, one of satisfaction, and two of both involvement and satisfaction. A previous wider review of shared decision making measures in 2007 identified 18 measures [60], including both measures selected in our study. The previous review concluded, as did ours, that psychometric evaluation is absent or poor for many measures, with a specific concern raised about validity. The focus on assessing convergent, divergent and predictive validity of CDIS addresses this issue.

The Satisfaction sub-scale was modified from the Satisfaction with Decision Scale [46]. The original scale was evaluated in a sample of 252 women making decisions about management of menopause and hormone replacement therapy (HRT). The scale had internal consistency of 0.88, principal component analysis indicated discriminant validity, and evidence for predictive validity relating to decision certainty and HRT use at 12-month follow-up. The comparability with the evaluation of CDIS indicates that modification has not substantially compromised psychometric adequacy.

### Strengths and limitations

The main strengths of this study are methodology and sample frame. The application of an established methodology for developing culturally valid translations of a patient-rated outcome measure has maximised the likelihood that CDIS data collected using any of the five languages will be both comparable and conceptually equivalent. The size of the sample, the involvement of six countries from across Europe, and the involvement of a routine sample of people using specialist mental health services in each country all increase the generalisability of the findings.

The primary limitations relate to the non-systematic review in Stage 1, and to the psychometric evaluation methodology. There was a relatively lower validation for CDIS service user rating of Involvement. Although it may be indicating that sub-scale to be less reliable or valid, the more positive findings from other sub-scales suggests that service user rated involvement appears to be a process which has other influences than either satisfaction or staff-rated involvement. Specifically, from the service user perspective, experience of involvement did not co-vary with other assessed variables, and in this study there was no gold standard independent rating of involvement which could be used as a comparator. The OPTION Scale is an observer-rated measure of patient involvement in decision-making, which has been used to investigate the extent to which psychiatrists involve service users in out-patient consultations [61]. A future approach for further investigating convergent validity for CDIS Involvement sub-scale might involve comparison with OPTION rating.

### Clinical and research implications

CDIS is the first short, standardised measure of involvement and satisfaction with a specific decision related to mental health care, which is suitable for use across a range of clinical settings and available in five languages. This measure will inform clinical practice and future research, particularly in relation to involvement in decision-making. Most staff would argue that increased service user satisfaction is positive. Indeed, mental health professionals would prefer to be evaluated in relation to satisfaction rather than clinical improvement [62]. We showed that CDIS Satisfaction scale can be analysed as either continuous or three-category data, since both show adequate validity. Whichever approach is used, our data are consistent with an interpretation that satisfaction with a decision-making process is relatively aligned with other process variables capturing aspects of the therapeutic alliance. Predictors of therapeutic alliance include age, gender, severity and kind of symptoms (positive, negative, disorganized), interpersonal factors, diagnosis, frequency of service contact, better awareness of treatment need, and illness insight [63-68]. It is therefore plausible that these also influence satisfaction with decision-making.

However, there might be a more mixed view among staff about whether increased service user involvement is always positive. Increased involvement is a priority for service users but not staff [69]. For example, a survey of 352 psychiatrists identified a differing emphasis on level of involvement for different patients (involvement less important when capacity reduced) and decision topic (more involvement endorsed for psychosocial decisions such as work, housing and psychotherapy, less for admission, medication, diagnostic procedures) [70]. This variability may be positive, reflecting the mature application of clinical judgement, or inter-professional differences, or varying levels of perceived responsibility for care between acutely unwell and less unwell service users. This last suggestion is consistent with our data, showing more active staff-rated involvement from more recovered service users, which has clinical implications for tailoring the balance of power in decision-making on the basis of stage of recovery. Or the variability may be negative, reflecting cognitive errors created through clinical training [71]. The CDIS measure is feasible for routine clinical use, and in identifying the level of involvement, provides a tool to support reflective practice by staff. For example, the association found in this study between staff-rated passive involvement by the service user and subsequent decision implementation is consistent with a paternalistic decision-making approach by staff leading to compliant but disempowered behaviours by service users, which may not optimise outcome.

In relation to research, the CDIS provides a tool for understanding the experience of a specific decision. This allows several types of research. First, to what extent is the experience consistent with pre-stated preferences [72], and does this matter? Second, how do characteristics of the worker, the service user, and the decision topic influence the decision-making experience? Third, how responsive is CDIS to capturing the impact of interventions to promote shared decision-making, and what change in CDIS rating constitutes clinically meaningful change? Fourth, and perhaps most importantly, do either of involvement or satisfaction predict decision implementation, and does implementation in turn predict outcome? All of these questions are being addressed in the CEDAR Study [2].

Shared decision-making is widely advocated in mental health services [5], and is feasible even in in-patient settings [7]. However, distinguishing between shared decision-making and sophisticated techniques of persuasion is not straightforward, Both staff [1] and service users [11] use approaches to influence the views of the other. There is preliminary evidence of benefit from shared decision-making in mental health for medication management [73,74]. However, although truly shared decision-making is already envisioned by some [38], the most recent Cochrane review was unable to find sufficient robust data to determine whether shared decision-making for people with mental health conditions is effective [10]. It is known that relationship are important in mental health, for example in in-patient settings [75], and more generally they support recovery [76]. CDIS data may contribute to the development of a stronger empirical underpinning of when, and why, high involvement of mental health service users in decision-making is beneficial.

## Abbreviations

CEDAR: Clinical decision making and outcome in routine care for people with severe mental illness (study acronym); CDIS: Clinical Decision-making Involvement and Satisfaction; CDIS-P: CDIS patient version; CDIS-S: CDIS staff version; CPS: Control Preference Scale; GAF: Global Assessment of Functioning; HAS: Helping Alliance Scale; HoNOS: Health of the Nation Outcome Scales; HRT: Hormone replacement therapy; ISPOR: International Society for Pharmacoeconomics and Outcomes Research; MANSA: Manchester Short Assessment; OQ-45: Outcome Questionnaire – 45; SCID: Structured Clinical Interview for DSM-IV; STORI: Stages of Recovery Inventory; SWD: Satisfaction With Decision-making scale; TAG: Threshold Assessment Grid.

## Competing interests

The authors declare that they have no competing interests.

## Authors’ contributions

All authors were responsible for the conception of the project. MS, KA, EC, DG, HJ and BP were involved in writing the manuscript, and all authors critically revised and approved the final manuscript.

## Pre-publication history

The pre-publication history for this paper can be accessed here:

http://www.biomedcentral.com/1472-6963/14/323/prepub
